# Macrophages Are Dispensable for Postnatal Pruning of the Cochlear Ribbon Synapses

**DOI:** 10.3389/fncel.2021.736120

**Published:** 2021-10-21

**Authors:** Chaorong Yu, Hui-Ming Gao, Guoqiang Wan

**Affiliations:** ^1^MOE Key Laboratory of Model Animal for Disease Study, Department of Otorhinolaryngology-Head and Neck Surgery, The Affiliated Drum Tower Hospital of Medical School, Model Animal Research Center of Medical School, Nanjing University, Nanjing, China; ^2^Jiangsu Key Laboratory of Molecular Medicine, Medical School of Nanjing University, Nanjing, China; ^3^Institute for Brain Sciences, Nanjing University, Nanjing, China; ^4^Research Institute of Otolaryngology, Nanjing, China

**Keywords:** ribbon synapse, synaptic pruning, cochlear macrophages, hair cells, CX3CR1

## Abstract

Ribbon synapses of cochlear hair cells undergo pruning and maturation before the hearing onset. In the central nervous system (CNS), synaptic pruning was mediated by microglia, the brain-resident macrophages, *via* activation of the complement system. Whether a similar mechanism regulates ribbon synapse pruning is currently unknown. In this study, we report that the densities of cochlear macrophages surrounding hair cells were highest at around P8, corresponding well to the completion of ribbon synaptic pruning by P8–P9. Surprisingly, using multiple genetic mouse models, we found that postnatal pruning of the ribbon synapses and auditory functions were unaffected by the knockout of the complement receptor 3 (CR3) or by ablations of macrophages expressing either LysM or Cx3cr1. Our results suggest that unlike microglia in the CNS, macrophages in the cochlea do not mediate pruning of the cochlear ribbon synapses.

## Introduction

Hearing involves neural transmission mediated by the ribbon synapses between the auditory hair cells and spiral ganglion neurons (SGNs). Ribbon synapses continue to mature after birth. At the end of the first postnatal week, the density of ribbon synapses reaches its highest level compared to those at adulthood or onset of hearing (around P12 in the mouse). Therefore, from P6 to P12, the immature synapses would either be enhanced or eliminated, leading to about a 50% reduction in the total number of synapses (Delacroix and Malgrange, 2015; Brown et al., 2017). This process, also termed synaptic pruning, involves neurite refinement and retraction of the immature SGN terminals (Barclay et al., 2011). The remaining ribbons undergo varied morphological and molecular changes to acquire a functional response to sound stimulation (Safieddine et al., 2012). As the ribbon synapses mature at the 2nd postnatal week, the Cav1.3-dependent Ca^2+^ current reduces, which contributes to increased exocytosis. Such maturation of synaptic exocytosis is important for encoding continuous and finely graded signals necessary for the temporal acuity and fidelity of acoustic information (Brandt et al., 2005; Frank et al., 2010; Delacroix and Malgrange, 2015).

Immuno-homeostasis within the mammalian cochlea is supported by cells of both innate and adaptive immune systems (Rai et al., 2020). Macrophages, the monocyte-derived phagocytes, are the major players of the cochlear innate immune system mediating pathogen detection and elimination, tissue homeostasis, and injury responses (Warchol, 2019). Macrophages have been found in various cochlear regions, including the sensory epithelia, spiral ligament, spiral limbus, Rosenthal’s canal, and stria vascularis (Dong et al., 2018). While the cochlear resident macrophages may be derived from either yolk sac hematopoiesis or the fetal liver (Shi, 2010; Kishimoto et al., 2019), bone marrow-derived macrophages can infiltrate cochlea upon injuries by noise exposure or ototoxicity (Wood and Zuo, 2017). Functions of macrophages were in part regulated by the complement system, which comprises over 35 cell-associated and soluble molecules, including C1q, mannan-binding lectin, ficolins, and their activation products C3, C4, C5 etc. (Bohlson et al., 2014). Activation of the complement receptors expressed on macrophages leads to modulation of cytokine production, inflammatory responses, and increased phagocytosis (Bohlson et al., 2014).

The molecular mechanisms for the pruning and maturation of ribbon synapses is unclear now. However, recent studies indicate that microglia, the brain-resident macrophages, play instructive roles in synaptic pruning of the central nervous system (CNS). It has been reported that progranulin deficiency in the CNS resulted in upregulation of lysosomal and innate immunity genes and increased complement production, which promoted synaptic pruning by complement-dependent microglial phagocytosis (Lui et al., 2016). Specifically, engulfment of presynaptic inputs by microglia in the postnatal retinogeniculate system was dependent on neural activity and the microglia-specific phagocytic signal pathway, complement receptor 3 (CR3)/C3 (Schafer et al., 2012). However, whether macrophages are involved in the pruning of cochlear ribbon synapses remains to be determined.

In this study, we applied genetic mouse models to specifically eliminate either the macrophages or complement receptor 3 and provided strong evidence that cochlear macrophages do not mediate ribbon synaptic pruning in the cochlea. Our results highlight the differential involvement of macrophages in the pruning of the central and peripheral synapses and point to a yet to be defined mechanism for the pruning and maturation of cochlear ribbon synapses.

## Materials and Methods

### Animal Models

Cx3cr1-CreER-IRES-EYFP (Cx3cr1-CreER-YFP) mice (Stock: 021160), Itgam-KO (CR3-KO) mice (Stock: 003991), Lyz2-Cre (LysM-Cre) mice (Stock: 004781), Rosa26-ACTB-mTdTomato-mGFP (mTmG) mice (Stock: 007676), Rosa26-DTA mice (Stock: 006331) were obtained from the Jackson Laboratory. Wildtype C57BL/6J and FVB/N mice were purchased from Gempharmatech Inc, China. Mice were injected intraperitoneally with tamoxifen (Sigma, T5648, dissolved in corn oil) daily at 33 mg/kg. Both male and female animals in mixed C57BL/6J and FVB/N backgrounds were used in this study. Animals were housed at the Model Animal Research Center of Nanjing University and were protected from overt noise exposure from the surrounding environment for the duration of the investigation. All experiments were approved by the Institutional Animal Care and Use Committee and carried out in accordance with the animal protocol of the Model Animal Research Center of Nanjing University (permit number #WGQ01).

### Cochlear Tissue Preparation

Cochlear tissues were harvested at two main time frames, P0-P12 and 3–4 weeks. After dissection, the cochleae were perfused gently through the oval window using a 1 ml syringe with 4% paraformaldehyde in PBS, pH 7.4, followed by post-fixation with the same fixative at room temperature for 2 h. Cochlear samples were then flushed three times with PBS and placed in 5% EDTA in PBS on the rocker at room temperature for 3–4 days for decalcification. Once fully decalcified, the specimens were washed in PBS twice for 10 min on the rocker.

### Cochlear RNA Extraction and RT-qPCR

Whole cochlear tissues from postnatal wildtype mice, CR3(+/–) or CR3(−/−) mice were used for total RNA isolation using Trizol reagent (Takara, 9108). Reverse transcription of total RNA was performed with the Primescript RT reagent kit (Takara, RR047A). Quantitative PCR was performed with the Hieff UNICON^®^ qPCR SYBR Green Master Mix (YEASEN, 11198ES03) on LightCycler 96 instrument (Roche). Details of the primers were as follows: GAPDH, forward primer 5’-ACC ACG AGA AAT ATG ACA ACT CAC-3’, reverse primer 5’-CCA AAG TTG TCA TGG ATG ACC-3’; C3, forward primer 5’-CAG GAT GGC GAT AAG AAG ATT-3’, reverse primer 5’-ATG ACA GTG ACG GAG ACA-3’; CR3, forward primer 5’-ATG ACT CTT AAA GCT CTT CTG GTC-3’, reverse primer 5’-TTA ACA GCC TTT GCC TCC T-3’. Data are normalized to GAPDH, and fold changes are calculated by using 2^−ΔΔCT^ method.

### Immunofluorescence

For cryo-section analysis, the inner ear tissues were embedded and frozen in OCT media (Sakura Finetek, Torrance, CA, USA), followed by cryo-sectioning at 14 μm thickness. The sections were dried at 37°C for 1 h, rinsed with PBS before subsequent immunostaining. For wholemount immunofluorescence, cochlear tissues were micro-dissected and permeabilized by freeze-thawing in 30% sucrose. The cry-sections or micro-dissected pieces were blocked in 5% normal horse serum (NHS) with 1% Triton X-100 in PBS for 1 h, followed by incubation in primary antibodies (diluted in blocking buffer) at 4°C for 16 h. The primary antibodies used in this study were anti-myosin VIIa (rabbit anti-Myo7a IgG, 25-6790, Proteus Biosciences, 1:500); anti-C-terminal binding protein 2 (mouse anti-CtBP2 IgG1, 612044, BD Biosciences, 1:200); anti-glutamate receptor 2 (mouse anti-GluA2 IgG2a, MAB397, Millipore, 1:2,000); anti-shank1a (rabbit anti-Shank1a IgG, RA19016, NEURONS, 1:1,000), anti-neurofilament H (chicken anti-NFH IgY, AB5539, Millipore, 1:1,000); anti-Iba1 (rabbit anti-Iba1 IgG, 019-19741, WAKO, 1:2,000). Secondary antibodies were Alexa Fluor 488, 568, or 647-conjugated goat antibodies (Life Technologies, USA).

### Confocal Microscopy

All pieces of each cochlea were imaged at low power to convert cochlear locations into frequencies using a custom ImageJ plugin^1^. Confocal z-stacks of the 5.6, 8, 11.3, 16, 22.6, and 32 kHz regions from each cochlea were imaged using a Zeiss LSM880 (Zeiss, Germany) or Leica TCS SP5II microscopes (Leica, Germany). Analyses of cochlear ribbon synapses were performed as previously described (Wan et al., 2014). ImageJ software (version 1.51j8, NIH, MD) was used for image processing and three-dimensional reconstruction of z-stacks.

### Mouse Auditory Phenotyping

Animals were anesthetized by intraperitoneal injections of xylazine (10 mg/kg) and ketamine (100 mg/kg), prior to auditory brainstem response (ABR) and distortion-product otoacoustic emission (DPOAE) tests. For ABR tests, three needle electrodes were inserted under the skin: one at the dorsal midline of the head between the two ears, one behind the left pinna, and one grounding electrode at the base of the mouse tail. ABR potentials were evoked with 5 ms tone pips (0.5 ms rise-fall, with a cos2 envelope, at 33/s) delivered to the eardrum at log-spaced frequencies from 5.6–32 kHz. The responses were amplified (10,000×) and filtered (0.3–3 kHz) with an analog-to-digital board in a PC-based data-acquisition system. Sound pressure levels (SPLs) were raised at 5 dB-step from 10–80 decibels (dB). At each level, 1,024 responses were averaged (with alternating polarity) after “artifact rejection”. The DPOAE signal in response to primary and secondary tones with frequencies f1 and f2 respectively was recorded at the third frequency (2 × f1) − f2, with f2/f1 = 1.2, and the f2 level 10 dB lower than the f1 level. SPLs at the ear canal were amplified and digitally sampled at 4 ms intervals. DPOAE thresholds were defined as the f1 level required to evoke a response at −10 dB SPL. Both DPOAE and ABR recordings were carried out using EPL cochlear function test suite software (Mass Eye and Ear, Boston, MA, USA). ABR peak 1 (P1) amplitudes, P1 latencies, P2 latencies, and P1-P2 inter-peak latencies were analyzed with ABR peak analysis software (Mass Eye and Ear), as previously reported (Wan and Corfas, 2017).

### Statistical Analyses

Statistical tests were carried out using Graphpad Prism 8 (Graphpad Software Inc., La Jolla, CA). Data points were reported as mean ± SD or SEM as specified in the figure legends. Statistical significance values were analyzed using unpaired Student’s *t*-test, one-way ANOVA, or two-way ANOVA as indicated in the figure legends.

## Results

### Cochlear Ribbon Synapses Undergo Dynamic Pruning After Birth

**Figure 1:**
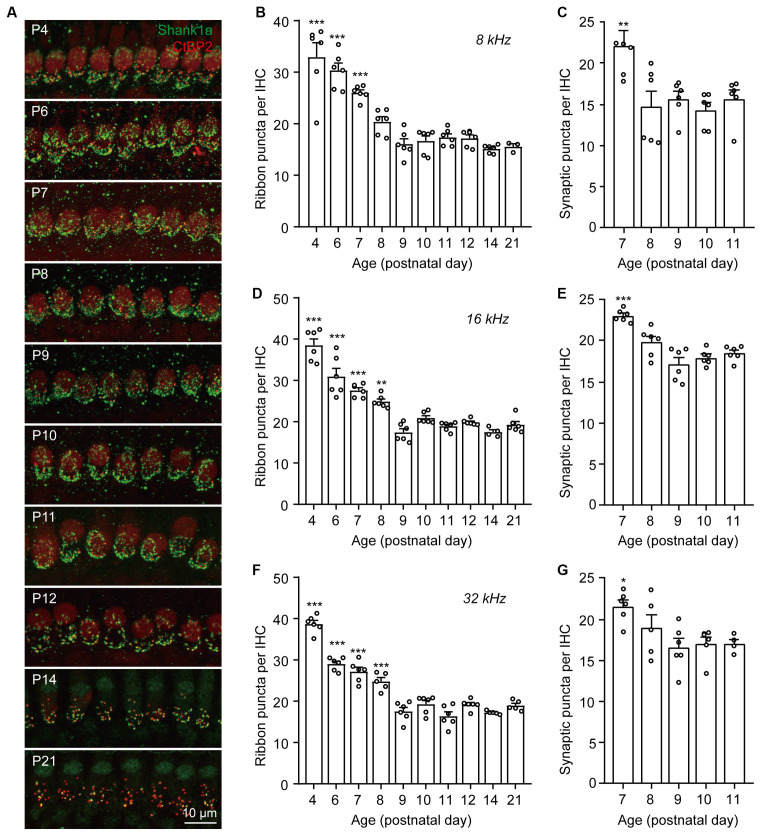
Dynamic changes of IHC ribbon synapse densities during postnatal development. **(A)** Representative images of presynaptic and postsynaptic specializations from inner hair cells at different postnatal ages. The images were taken at the middle cochlear frequency (16 kHz). Synapses within the entire length of the images were counted. **(B–G)** Quantitative analyses of synaptic ribbon counts **(B,D,F)** and putative ribbon synapses **(C,E,G)** at 8 kHz **(B,C)**, 16 kHz **(D,E)**, and 32 kHz **(F,G)** cochlear regions. *N* = 3–6, error bars represent mean ± SEM. **(B,D,F)** **p < 0.01 and ***p < 0.001 comparing to data at P21; **(C,E,G)** *p < 0.05, **p < 0.01 and ***p < 0.001 comparing to data at P11 by one-way ANOVA.

To investigate the dynamics of synaptic pruning and maturation after birth, F1 progenies (generated from C57BL/6J and FVB/N mating pairs) aged at postnatal days (P) 4, 6, 7, 8, 9, 10, 11, 12, 14, and 21 were used for synaptic counts. Pre-synaptic ribbons and post-synaptic structures were labeled with CtBP2 and Shank1a, respectively. Consistent with previous reports (Huang et al., 2012; Michanski et al., 2019), extensive synaptic pruning and refinement were observed in the postnatal cochlea (Figure 1A). Quantitative analyses of both presynaptic ribbons and putative synapses (co-localized CtBP2 and Shank1a) suggested that synaptic pruning was mostly completed by P7–P8 along the cochlear turns (Figures 1B–G). However, although the synaptic density was reduced to the same level as that in the mature cochlea by P8, the spatial orientation and refinement continued to undergo dynamic maturation from P8 to P14 (Figure 1A). These results indicate that while cochlear synapses continued to refine and mature until hearing onset, the pruning of ribbon synapses was largely completed by P8 in the mouse cochlea.

### Dynamic Changes of the Postnatal Cochlear Macrophages

**Figure 2:**
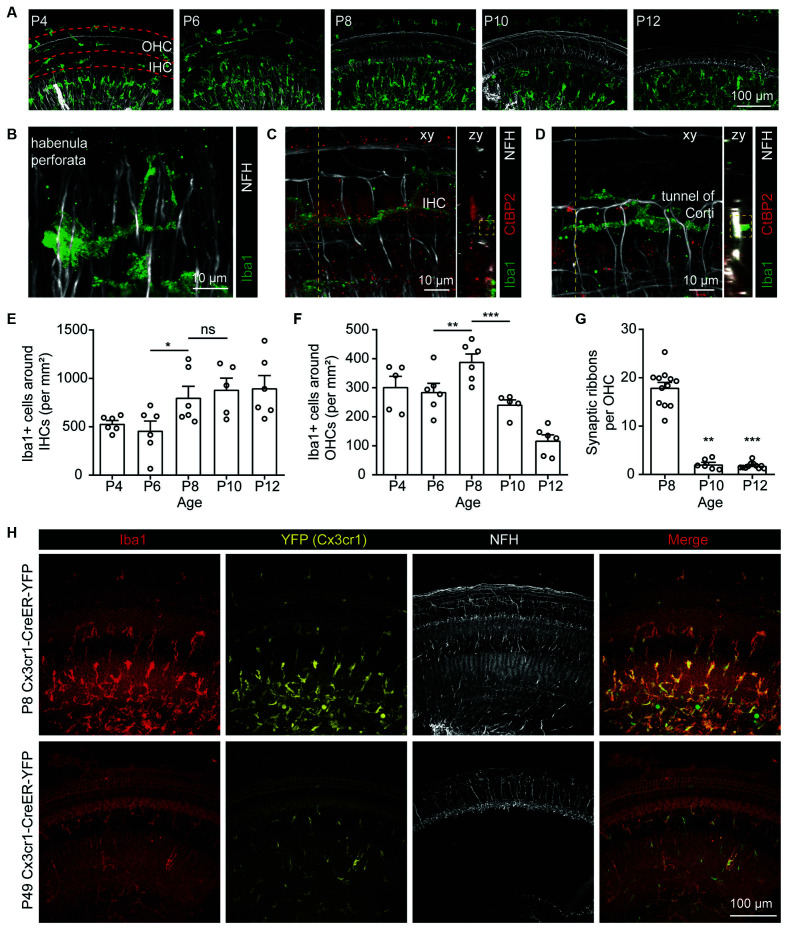
Localizations of macrophages in the postnatal cochlea.** (A)** Dynamic localization of macrophages in P4, P6, P8, P10, P12 cochleae. Dashed red lines indicate regions around OHCs or IHCs. **(B)** Macrophages surrounding spiral neuron axons in proximity to the habenula perforata. **(C,D)** Macrophages localized around the **(C)** IHCs and **(D)** tunnel of Corti. Dotted lines indicate positions for zy projections. Dotted boxes show colocalizations of macrophagic processes with **(C)** pre-synaptic ribbon or **(D)** nerve terminal. **(E,F)** Quantification of the dynamic changes in the densities of Iba1+ macrophages around IHCs **(E)** and OHCs **(F)** from P4 to P12. *N* = 5–6, error bars represent mean ± SEM. ns: not significant, *p < 0.05, **p < 0.01 and ***p < 0.001 by one-way ANOVA. **(G)** Quantification of the dynamic changes in the number of OHC synaptic ribbons from P8 to P12. *N* = 6–12, error bars represent mean ± SEM. **p < 0.01 and ***p < 0.001 by one-way ANOVA. **(H)** Reduced density of macrophages in the adult cochlea. The Cx3cr1-driven YFP (yellow) co-localized with Iba1 immunostaining in cochlear macrophages (red).

To investigate whether cochlear macrophages were involved in ribbon synaptic pruning, we collected cochleae at P4, P6, P8, P10, P12, and immunolabeled macrophages and SGN neurites with Iba1 and NFH, respectively (Figure 2A). Macrophages were observed throughout the cochlear sensory epithelia, including the organ of Corti and spiral lamina (Figure 2A). Particularly, Iba1+ macrophages were found to wrap the auditory nerve fibers beneath the habenula perforata (Figure 2B) as well as in close contact with the synaptic ribbons (Figure 2C) and the nerve terminals (Figure 2D). We then counted the macrophages in the inner hair cell (IHC) region and found that the densities of macrophages increased significantly from P6 to P8 (Figure 2E), coincident with the completion of synaptic pruning at P8-P9 (Figure 1). Similarly, the number of macrophages around the outer hair cells (OHCs; Figure 2F) and OHC synaptic ribbons (Figure 2G) were also highest at P8. Cochlear macrophages reduced in number gradually after P8 and were scarcely localized in the adult cochlea (Figure 2H). The proximal and dynamic correlations between the macrophages and synaptic structures suggest that macrophages may potentially be involved in the pruning and refinement of cochlear ribbon synapses and nerve terminals.

### Ablation of Cochlear Macrophages Did Not Affect Auditory Function and Ribbon Synapses Pruning

**Figure 3:**
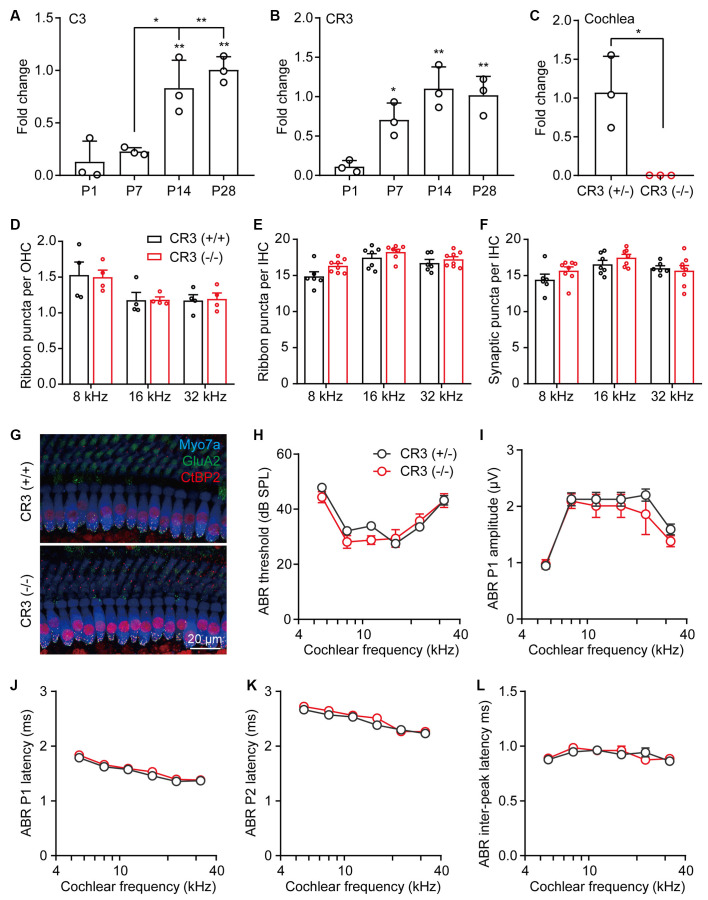
Complement receptor CR3 was not required for pruning of cochlear ribbon synapses and auditory function. **(A,B)** RT-qPCR analyses of **(A)** C3 or **(B)** CR3 expression in P1, P7, P14, and P28 cochleae. *N* = 3, error bars represent mean ± SD. *p < 0.05 and **p < 0.01 by one-way ANOVA. **(C)** RT-qPCR analyses of CR3 knockout in the cochlea. *N* = 3, error bars represent mean ± SD. *p < 0.05 by unpaired Student’s *t*-test. **(D,E)** Quantitative analyses of OHC **(D)** and IHC **(E)** synaptic ribbons in P21 wildtype and CR3 knockout cochlea. **(F)** Quantitative analyses of putative IHC ribbon synapses (colocalized GluA2 and CtBP2 signals) in P21 wildtype and CR3 knockout cochlea. *N* = 4–8, error bars represent mean ± SEM. **(G)** Representative confocal images of P21 wildtype and CR3 knockout cochlea at 16 kHz. Myo7a, hair cells; GluA2, postsynaptic receptors, and CtBP2, pre-synaptic ribbons. **(H–L)** ABR tests of P21 wildtype and CR3 knockout mice. **(H)** ABR thresholds, **(I)** ABR P1 amplitudes, **(J)** ABR P1 latencies, **(K)** ABR P2 latencies, and **(L)** ABR P1-P2 inter-peak latencies. ABR amplitudes and latencies were analyzed at 80 dB SPL. *N* = 8 (CR3 KO) or 14 (wildtype), error bars represent mean ± SEM.

Synaptic pruning in the CNS is dependent on neural activity and the microglia-specific phagocytic signaling pathway, complement 3 (C3), and its receptor CR3 (Schafer et al., 2012). Using RT-qPCR, we found that both C3 and CR3 showed temporal increases in the postnatal cochleae, where the expression of C3 increased significantly from P7 to P14 (Figure 3A) and the expression of CR3 showed a marked increase from P1 to P7 (Figure 3B). These results suggest that both C3 and CR3 were dynamically expressed and may play a physiological role in the postnatal cochlea. To investigate the role of C3/CR3 in cochlear synaptic pruning, we analyzed the changes of ribbon synapses in the CR3 knockout mice. Complete knockout of CR3 was validated by RT-qPCR in the CR3(−/−) cochlea (Figure 3C). Unexpectedly, densities of both pre-synaptic ribbons and putative ribbon synapses were normal in the CR3(−/−) mice (Figures 3D–G). In addition, auditory functions of the CR3-deficient mice were unchanged compared to the littermate controls (Figures 3H–L). These results suggest that complement receptor CR3 was not involved in cochlear synaptic pruning.

An interesting possibility is that macrophages may regulate ribbon synaptic pruning in a CR3-independent mechanism, as previously discussed (Warchol, 2019). Therefore, we ablated macrophages by expressing Diphtheria toxin fragment A (DTA) in LysM-expressing cells. LysM-Cre drives recombination in myeloid cell lineage, including monocytes, mature macrophages, and granulocytes (Clausen et al., 1999). The efficiency of recombination in the cochlea was first verified in P8 LysM-Cre:mTmG mice, which showed about 50% recombination of cochlear macrophages labeled with Iba1 (Supplementary Figure 1A). We then crossed LysM-Cre mice with Rosa-DTA mice to specifically and persistently ablate the macrophages. Interestingly, partial ablation of cochlear macrophages did not affect the synaptic density at P12 in the LysM-Cre:Rosa-DTA mice (Supplementary Figure 1B–D). In addition, the auditory functions, including DPOAE thresholds, ABR thresholds, P1 amplitudes, P1 latencies, P2 latencies, and P1-P2 inter-peak latencies were also normal in the adult LysM-Cre:Rosa-DTA mice (Supplementary Figure 1E–J). This result indicates that partial but constitutive ablation of cochlear macrophages did not affect ribbon synapse pruning and auditory function.

**Figure 4:**
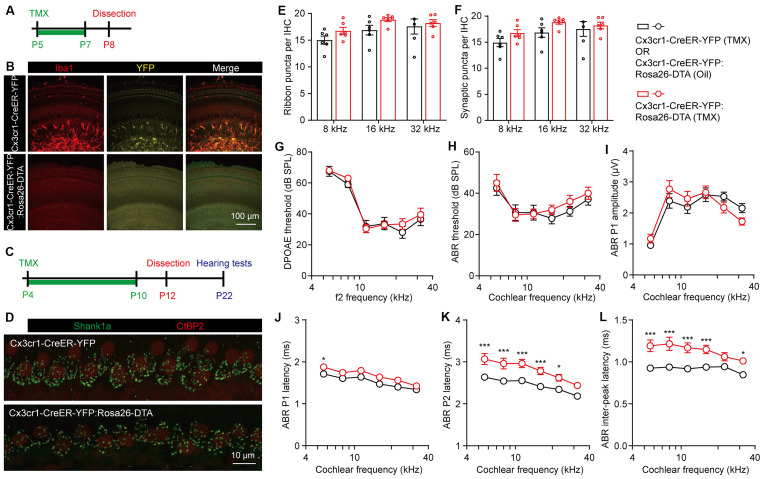
Temporary complete elimination of postnatal macrophages did not affect ribbon synapses pruning and auditory function. **(A,B)** Schematic diagram **(A)** and confocal images **(B)** showing conditional elimination of postnatal macrophages in Cx3cr1-CreER-YFP:Rosa26-DTA (macrophage-deficient) cochleae at 16 kHz region. **(C,D)** Schematic diagram **(C)** and confocal images **(D)** showing effects of postnatal macrophage elimination on cochlear ribbon synapses. Presynaptic ribbons and postsynaptic structures on P12 mice at 16 kHz were immunolabeled with CtBP2 (red) and Shank1a (green), respectively. **(E,F)** Quantitative analyses of IHC synaptic ribbons **(E)** and putative ribbon synapses **(F)** in P12 control and macrophage-deficient cochleae. *N* = 4–6, error bars represent mean ± SEM. **(G–L)** DPOAE thresholds **(G)**, ABR thresholds **(H)**, ABR P1 amplitudes **(I)**, ABR P1 latencies **(J)**, ABR P2 latencies **(K)**, and ABR P1-P2 inter-peak latencies **(L)** of P22 control and macrophage-deficient mice. Cx3cr1-CreER-YFP:Rosa26-DTA mice were injected with tamoxifen (TMX) from P4 to P10. Controls mice were either Cx3cr1-CreER-YFP mice injected with TMX or Cx3cr1-CreER-YFP:Rosa26-DTA injected with corn oil. *N* = 10 (TMX) or 16 (corn oil), error bars represent mean ± SEM. *p < 0.05 and ***p < 0.001 by two-way ANOVA.

To better eliminate the cochlear macrophages in a controlled manner, we then crossed Rosa-DTA with Cx3cr1-CreER-YFP mice, which allows tamoxifen-inducible recombination and expression of DTA in macrophages and microglial cells (Parkhurst et al., 2013). Cx3cr1-dependent YFP labeling was validated by co-immunostaining with Iba1, while DTA-mediated ablation of cochlear macrophages was achieved in Cx3cr1-CreER-YFP:Rosa26-DTA mice by tamoxifen injections (Figures 4A,B). As the cochlear ribbon synapses underwent dynamic regulation from P4 to P10 (Figure 1), we ablated the macrophages at this critical period by daily tamoxifen injections from P4 to P10, followed by synaptic counts at P12 and auditory function tests at P22 (Figure 4C). Similarly, we found that the pruning of ribbon synapses in Cx3cr1-CreER-YFP:Rosa26-DTA mice was also unaffected (Figures 4D–F). As almost all presynaptic ribbons were associated with adjacent postsynaptic Shank1 patches, counts of ribbon puncta (Figure 4E) and co-localized synaptic puncta (Figure 4F) were highly corroborative. DPOAE thresholds, ABR thresholds, and P1 amplitudes were also normal in macrophage-depleted mice (Figures 4G–I). Intriguingly, unlike CR3(−/−) or LysM-Cre:Rosa-DTA mice, complete ablation of macrophages from P4 to P10 resulted in prolonged ABR P1, P2, and P1-P2 inter-peak latencies (Figures 4J–L). This result is consistent with a previous report that cochlear macrophages regulated glial homeostasis, SGN myelination, and nerve conduction (Brown et al., 2017).

In summary, we provide compelling evidence that cochlear macrophages were not required for pruning of the postnatal ribbon synapses.

## Discussion

Ribbon synapses play a crucial role in transmitting sound-evoked signals from the sensory hair cells to the SGNs. Degeneration of the ribbon synapses underlies age-related and noise-induced sensorineural hearing loss, a pathology termed cochlear synaptopathy (Wan and Corfas, 2015). Similar to the CNS synapses, cochlear ribbon synapses also underwent postnatal pruning and maturation (Huang et al., 2012; Michanski et al., 2019). Understanding the cellular and molecular basis of ribbon synapse pruning may help identify strategies for regeneration and functional restoration of the cochlear synapses. In this study, we found that the maximal density of cochlear macrophages corresponds well to the completion of ribbon synaptic pruning at P8-P9. Surprisingly, unlike the critical role of microglia in CNS synapse pruning, the cochlear macrophages are not involved in the postnatal pruning of ribbon synapses.

Based on previous reports, functional and structural maturation of ribbon synapses took place at around P6 to P12 in mice (Huang et al., 2012; Michanski et al., 2019). By interrogating the dynamics of ribbon synapses at higher temporal resolution, we found that synaptic pruning proceeds from P4 to P8 and completes at P8-P9. This refined time course provides important temporal information for the identification of potential mechanisms involved in synaptic pruning.

In the CNS, postnatal synaptic pruning was primarily mediated by microglia, the brain-resident macrophages, *via* activation of the C3 complement system (Schafer et al., 2012; Lui et al., 2016). Since macrophages are also localized to the postnatal and adult cochlea, it has been hypothesized that the macrophages may promote the pruning of postnatal cochlear ribbon synapses in C3 complement-dependent manner (Coate et al., 2019; Warchol, 2019). Indeed, we found that both C3 and CR3 were dynamically expressed in the postnatal cochlea and that the densities of the cochlear macrophages in proximity to the hair cells were highly correlated with the reduction of ribbon synapses, with apparent colocalizations of both processes from macrophages and nerve fibers. Despite such remarkable correlation, deletion of the complement receptor CR3 or elimination of cochlear macrophages did not seem to affect the synaptic density or auditory function, excluding the involvement of macrophages in postnatal ribbon synapse pruning.

In addition to C3, other complement proteins, including C1q, C1ql1, and C4, were also implicated in the pruning of CNS synapses (Stevens et al., 2007; Kakegawa et al., 2015; Sekar et al., 2016). C1q in CNS neurons can be induced by immature astrocytes and also participated in synaptic elimination (Stevens et al., 2007). C1ql1, a member of the C1q family of proteins, was produced by climbing fibers of the motor cerebellum to mediate selective elimination of nerve fibers and synapses (Kakegawa et al., 2015). C4 is a schizophrenia risk gene in humans and also mediates synapse elimination during postnatal development in mice (Sekar et al., 2016). However, the involvements of C1q and C1ql1 in postnatal pruning of cochlear ribbon synapses have been excluded in knockout mouse models (Calton et al., 2014; Qi et al., 2021). While C4 is also expressed in the postnatal cochlea and is induced by Slc26a4 deficiency, whether it plays a role in cochlear synapse pruning remains to be determined (Jabba et al., 2006).

Nevertheless, functional contributions of cochlear macrophages to other aspects of the peripheral auditory system cannot be overlooked. Dendrites of SGNs, similar to ribbon synapses, were also eliminated during postnatal development, likely *via* sequential retractions of the immature SGN processes (Druckenbrod and Goodrich, 2015). Interestingly, macrophages appeared to play a homeostatic role by engulfing glial cells and the fragments of auditory nerve fibers (Brown et al., 2017), resulting in altered nerve conduction velocities, as we have also observed. Macrophages play even more prominent roles in the cochlea following insults, such as noise exposure or ototoxicity (Warchol, 2019). Immune cells would be recruited to cochleae following acoustic trauma (Tornabene et al., 2006) and bone marrow-derived macrophages would migrate to the spiral ligament, perilymphatic compartment walls, and limbus regions (Tan et al., 2008). Recruitment of the Cx3cr1-expressing macrophages to the cochleae was likely mediated by the fractalkine signaling (Kaur et al., 2015; Sun et al., 2015; Yang et al., 2015; Mizushima et al., 2017), although Sato et al. (2010) reported that the absence of Cx3cr1 would lead to more macrophages migrating to the cochleae after kanamycin exposure.

The precise roles of macrophages in the injured cochleae are two folds. When cochleae were exposed to ototoxic drugs or noise stimulation, macrophages would induce hair cell loss and impair auditory function (Sato et al., 2010; Sun et al., 2015; Yang et al., 2015; Mizushima et al., 2017). However, it appears that the cochlear resident macrophages also could protect hair cells from the damaging effects of the infiltrating immune cells (Wood and Zuo, 2017). Furthermore, macrophages could promote SGN survival after selective hair cell lesion (Kaur et al., 2015, 2018). Most importantly, expression of Cx3cr1 in macrophages was required for spontaneous recovery of ribbon synapses after moderate noise trauma in C57BL/6 mice (Kaur et al., 2019). It is now evident that macrophages may play distinct and context-dependent roles in cochlear pathophysiology. Our data suggest that cochlear macrophages are not required for postnatal pruning of ribbon synapses; whether and how these macrophages are involved in myelination, neuronal activity and other aspects of cochlear plasticity remains to be determined.

While the cellular basis of postnatal cochlear ribbon synapses remains elusive, a couple of signaling pathways were reported to affect ribbon synaptic plasticity. For example, in the postnatal cochlea, Neurotrophin-3 (Ntf3) was required for the formation and maintenance of ribbon synapses. Ntf3 derived from supporting cells also promoted ribbon synapse regeneration after acoustic trauma (Wan et al., 2014; Wan and Corfas, 2015). In addition, thyroid hormone was necessary for synaptic pruning and maturation, while dispensable for synapse formation (Sendin et al., 2007; Ng et al., 2013; Delacroix and Malgrange, 2015; Sundaresan et al., 2016). As thyroid hormone receptor α was expressed in both SGNs and IHCs in rats (Lautermann and Ten Cate, 1997), it is possible that thyroid hormone could mediate ribbon synaptic pruning by directly regulating genes expressions in hair cells and SGNs. In the absence of thyroid hormone signaling, the number of morphologically immature synapses was increased. Such defect was associated with impairment of IHC differentiation, which prevented upregulation of the BK potassium channels required to cease the prehearing spontaneous activity (Delacroix and Malgrange, 2015; Sundaresan et al., 2016). However, whether these signaling pathways play deterministic roles in structural and functional maturation of the cochlear ribbon synapses remains to be investigated.

## Data Availability Statement

The raw data supporting the conclusions of this article will be made available by the authors, without undue reservation.

## Ethics Statement

The animal study was reviewed and approved by the Institutional Animal Care and Use Committee of Model Animal Research Center of Nanjing University.

## Author Contributions

GW conceived the study. CY and GW designed the experiments and wrote the manuscript. CY performed the experiments and analyzed the results. H-MG provided experimental resources. All authors contributed to the article and approved the submitted version.

## Conflict of Interest

The authors declare that the research was conducted in the absence of any commercial or financial relationships that could be construed as a potential conflict of interest.

## Publisher’s Note

All claims expressed in this article are solely those of the authors and do not necessarily represent those of their affiliated organizations, or those of the publisher, the editors and the reviewers. Any product that may be evaluated in this article, or claim that may be made by its manufacturer, is not guaranteed or endorsed by the publisher.

## Footnotes

^1^https://myfiles.meei.harvard.edu/xythoswfs/webui/_xy-e693768_1-t_wC4oKeBD.

## Supplementary Materials

SUPPLEMENTARY FIGURE 1 Persistent partial elimination of macrophages did not affect ribbon synapses pruning and auditory function. **(A)** A subset of cochlear macrophages was labeled by crossing LysM-Cre with mTmG mice. mGFP (green), Iba1 (red). **(B)** Representative confocal images of P12 LysM-Cre control and LysM-Cre:Rosa26-DTA cochlear ribbon synapses at 16 kHz. **(C,D)** Quantitative analyses of IHC synaptic ribbons **(C)** and putative ribbon synapses **(D)** in P12 LysM-Cre control and LysM-Cre:Rosa26-DTA cochleae. *N* = 4–6, error bars represent mean ± SEM. **(E–J)** DPOAE thresholds **(E)**, ABR thresholds **(F)**, ABR P1 amplitudes **(G)**, ABR P1 latencies **(H)**, ABR P2 latencies **(I)**, and ABR P1-P2 inter-peak latencies **(J)** of P22 LysM-Cre control and LysM-Cre:Rosa26-DTA mice. *N* = 10 (LysM-Cre:Rosa26-DTA) or 12 (LysM-Cre control), error bars represent mean ± SEM.
