# Supplementary figures and images for: Macrophages Are Dispensable for Postnatal Pruning of the Cochlear Ribbon Synapses
Source: Front Cell Neurosci. 2021 Oct 21;15:736120. doi: 10.3389/fncel.2021.736120 (PMC8566810; doi:10.3389/fncel.2021.736120)

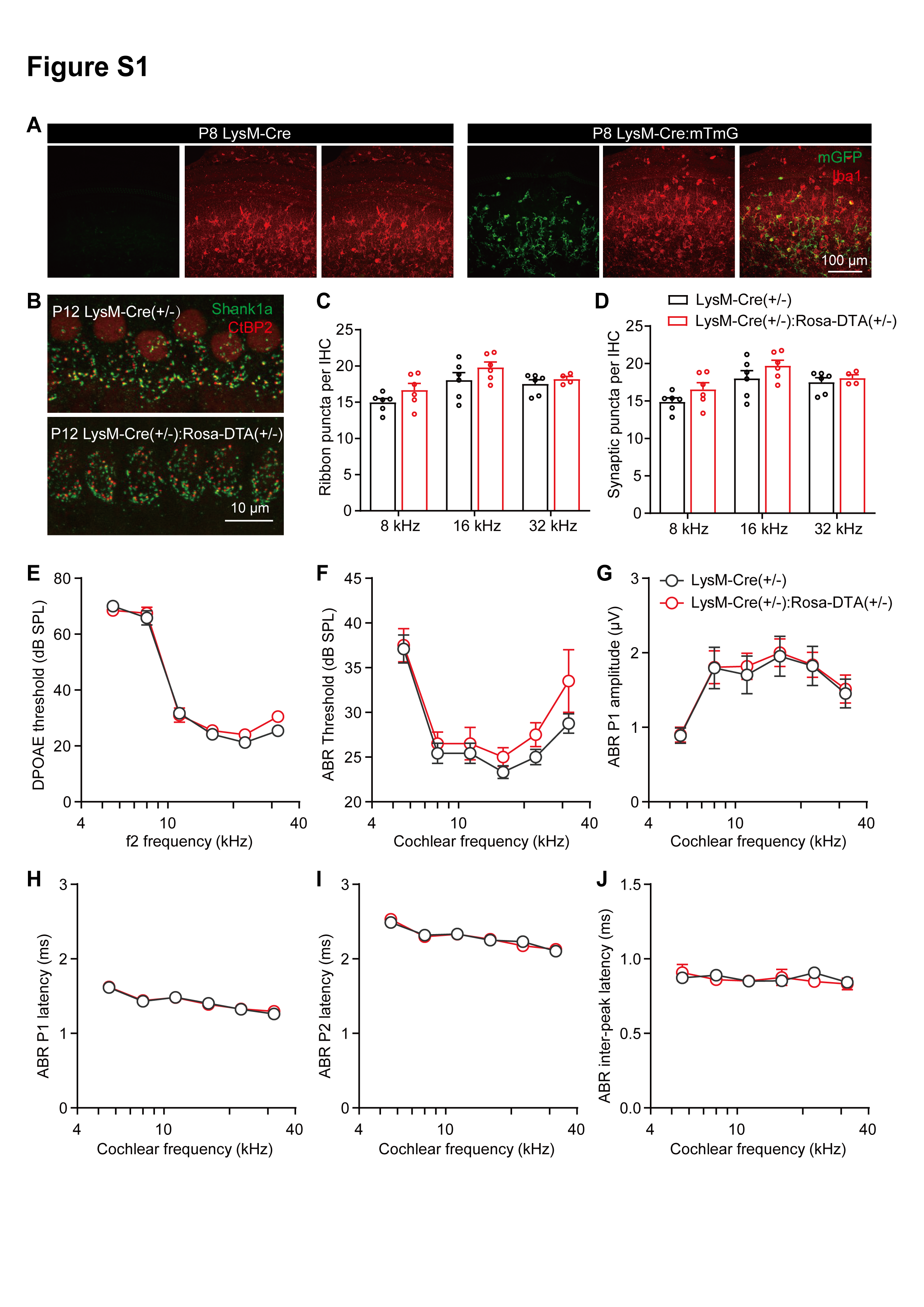

Supplement: SUPPLEMENTARY FIGURE 1 — Persistent partial elimination of macrophages did not affect ribbon synapses pruning and auditory function. (A) A subset of cochlear macrophages was labeled by crossing LysM-Cre with mTmG mice. mGFP (green), Iba1 (red). (B) Representative confocal images of P12 LysM-Cre control and LysM-Cre:Rosa26-DTA cochlear ribbon synapses at 16 kHz. (C,D) Quantitative analyses of IHC synaptic ribbons (C) and putative ribbon synapses (D) in P12 LysM-Cre control and LysM-Cre:Rosa26-DTA cochleae. N = 4–6, error bars represent mean ± SEM. (E–J) DPOAE thresholds (E), ABR thresholds (F), ABR P1 amplitudes (G), ABR P1 latencies (H), ABR P2 latencies (I), and ABR P1-P2 inter-peak latencies (J) of P22 LysM-Cre control and LysM-Cre:Rosa26-DTA mice. N = 10 (LysM-Cre:Rosa26-DTA) or 12 (LysM-Cre control), error bars represent mean ± SEM. [file Image_1.TIF]
